# (Photo)convert to pooled visual screening

**DOI:** 10.15252/msb.20209640

**Published:** 2020-06-16

**Authors:** Elena Ivanova, Anton Khmelinskii

**Affiliations:** ^1^ Institute of Molecular Biology (IMB) Mainz Germany

**Keywords:** Methods & Resources

## Abstract

Pooled genetic screening is a powerful method to systematically link genotype to phenotype and gain insights into biological processes, but applying it to visual phenotypes such as cell morphology or protein localization has remained a challenge. In their recent work, Fowler and colleagues (Hasle *et al*, 2020) describe an elegant approach for high‐throughput cell sorting according to visual phenotypes based on selective photoconversion. This allows combining the advantages of high‐content phenotyping by fluorescence microscopy with the efficiency of pooled screening to dissect complex phenotypes.

## Graphical abstract

The application of pooled genetic screens to link genotypes to visual phenotypes has remained a challenge. In their recent work, Fowler and colleagues describe an approach for high‐throughput cell sorting based on visual phenotypes in cells.

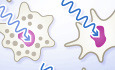

How is intracellular organization reshaped in response to genetic perturbations? How does the primary sequence of a protein determine its subcellular localization? What are the sources of cellular heterogeneity in response to infections or drug treatments? High‐content screening, which consists of high‐throughput microscopy and automated image analysis, provides a compelling way to address such questions (Boutros *et al*, 2015; Mattiazzi Usaj *et al*, 2016).

A typical high‐content screen is performed in an arrayed format such that different perturbations are tested in parallel but in separate wells in the array. This approach has yielded major insights into cellular organization and function in different model systems (Mattiazzi Usaj *et al*, 2016). But it is both costly and laborious, making it challenging to apply to a large number (10^4^–10^6^) of perturbations.

In contrast, screens performed in a pooled format are considerably more cost‐effective and high throughput. In this format, genetic perturbations are applied as a pool and cells with the desired phenotype are subsequently selected. For that, the phenotype of interest is typically linked to a selectable readout such as cell viability or expression of a fluorescent reporter, which can be used to collect the desired cell population using fluorescence‐activated cell sorting. Targeted DNA or RNA sequencing is then used to identify the perturbations enriched in the selected cells.

Applications of pooled screening have increased dramatically over the last 10 years. This is particularly prominent with genetic screens using clustered regularly interspaced short palindromic repeats (CRISPR), which rely on Cas proteins and guide RNAs to interfere with gene function in high throughput (Shalem *et al*, 2015; Hanna & Doench, 2020), and with deep mutational scanning (DMS) experiments, designed to determine the functional consequences of sequence variation (Fowler & Fields, 2014). Both types of experiments take advantage of inexpensive synthesis of pooled oligonucleotide libraries, which are used to generate libraries of guide RNAs required for CRISPR screens or libraries of sequence variants that form the basis of a DMS experiment. However, applying pooled screening to phenotypes such as cell or organelle morphology, or even protein localization, has not been trivial due to the difficulty of linking visual phenotypes to an easily selectable readout.

Recently, various approaches that enable high‐content pooled screens, while completely bypassing the need to link visual phenotypes to a selectable readout, have been developed. Some of these rely on sequential hybridization of fluorescent oligonucleotide probes (FISH) (Chen *et al*, 2015; Emanuel *et al*, 2017; Eng *et al*, 2019; Wang *et al*, 2019) or use *in situ* sequencing (Lee *et al*, 2014; Feldman *et al*, 2019) to read out genetic perturbations on single‐cell level following imaging‐based phenotyping. Another important advance was the development of an instrument capable of image‐activated cell sorting (IACS), which combines high‐throughput imaging with real‐time image analysis and cell sorting (Nitta *et al*, 2018). However, despite their potential, these methods are difficult to implement because they involve complex methodology or custom‐built equipment.

To address these limitations, in their recent study Hasle *et al* (2020) developed a new approach called visual cell sorting. Here, cells are first engineered to express a photoconvertible fluorescent protein, such as Dendra2, that will serve as a phenotypic marker (Fig 1). Following automated imaging and image analysis, the microscope is directed to photoconvert Dendra2 from the green‐fluorescent to the red‐fluorescent state specifically in cells with the desired phenotype. This procedure is repeated for each field of view, and the entire cell population is subsequently sorted according to the photoconversion state using fluorescence‐activated cell sorting (Fig 1). Notably, cells can be separated into up to four predefined phenotypic bins by modulating the extent of photoconversion. The authors applied visual cell sorting to perform a DMS analysis of a nuclear localization signal (NLS), which led to an improved NLS predictor and an NLS variant with greatly enhanced nuclear localization that should be a useful building block in synthetic biology applications. In a second demonstration of visual cell sorting, Hasle *et al* dissected cellular heterogeneity in response to the microtubule‐stabilizing drug paclitaxel by single‐cell RNA sequencing, which led to the identification of genes associated with paclitaxel resistance in chemotherapy.

**Figure 1:**
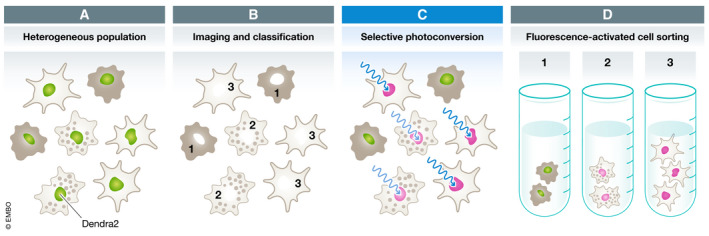
Visual cell sorting enables physical separation of a cell population according to visual phenotypes such as cell morphology or protein localization Cells expressing nuclear Dendra2 (green) and exhibiting different phenotypes (gray).Following imaging by microscopy, cells are automatically classified according to their phenotype (1, 2, or 3).Dendra2 is then photoconverted from the green to the red state, either partially (light magenta) or completely (magenta), specifically in cells with the desired phenotypes. The procedure is repeated for every field of view.Finally, cells are physically separated by fluorescence‐activated cell sorting into phenotypic bins using Dendra2 fluorescence as a marker.

Several features make visual cell sorting an attractive approach. First, it makes use of readily available instrumentation: a widefield fluorescence microscope, equipped with a laser and a digital micromirror device for fast photoconversion, and a fluorescence‐activated cell sorter. Second, with a throughput of ~ 4 cells/s (although below the speed of ~ 100 cells/s in IACS (Nitta *et al*, 2018)), in principle up to ~ 10^4^–10^5^ perturbations can be analyzed with this method, making it suitable for large visual DMS experiments or visual CRISPR screens. When using visual cell sorting with living cells, it is important to consider dilution of the photoconverted signal over time due to cell division. Especially in rapidly dividing organisms such as bacteria and yeast, the solution could involve analysis of fixed cells with a fixation‐resistant photoconvertible fluorescent protein (Paez‐Segala *et al*, 2015). Overall, visual cell sorting is an exciting addition to the high‐content screening toolbox that combines the power of pooled screens with the deep phenotyping of high‐content imaging and makes visual pooled screening using CRISPR, DMS, and other approaches generally accessible.
